# Bacterial Communities in Semen from Men of Infertile Couples: Metagenomic Sequencing Reveals Relationships of Seminal Microbiota to Semen Quality

**DOI:** 10.1371/journal.pone.0110152

**Published:** 2014-10-23

**Authors:** Shun-Long Weng, Chih-Min Chiu, Feng-Mao Lin, Wei-Chih Huang, Chao Liang, Ting Yang, Tzu-Ling Yang, Chia-Yu Liu, Wei-Yun Wu, Yi-An Chang, Tzu-Hao Chang, Hsien-Da Huang

**Affiliations:** 1 Institute of Bioinformatics and Systems Biology, National Chiao Tung University, Hsinchu, Taiwan; 2 Department of Biological Science and Technology, National Chiao Tung University, Hsinchu, Taiwan; 3 Department of Obstetrics and Gynecology, Hsinchu Mackay Memorial Hospital, Hsinchu, Taiwan; 4 Mackay Junior College of Medicine, Nursing and Management, Taipei, Taiwan; 5 Department of Medicine, Mackay Medical College, New Taipei City, Taiwan; 6 Health GeneTech Corporation, Taoyuan, Taiwan; 7 Graduate Institute of Biomedical Informatics, Taipei Medical University, Taipei, Taiwan; 8 Department of Biomedical Science and Environmental Biology, Kaohsiung Medical University, Kaohsiung, Taiwan; 9 Center for Bioinformatics Research, National Chiao Tung University, HsinChu, Taiwan; Institution and Department: Agricultural Research Service, United States of America

## Abstract

Some previous studies have identified bacteria in semen as being a potential factor in male infertility. However, only few types of bacteria were taken into consideration while using PCR-based or culturing methods. Here we present an analysis approach using next-generation sequencing technology and bioinformatics analysis to investigate the associations between bacterial communities and semen quality. Ninety-six semen samples collected were examined for bacterial communities, measuring seven clinical criteria for semen quality (semen volume, sperm concentration, motility, Kruger's strict morphology, antisperm antibody (IgA), Atypical, and leukocytes). Computer-assisted semen analysis (CASA) was also performed. Results showed that the most abundant genera among all samples were *Lactobacillus* (19.9%), *Pseudomonas* (9.85%), *Prevotella* (8.51%) and *Gardnerella* (4.21%). The proportion of *Lactobacillus* and *Gardnerella* was significantly higher in the normal samples, while that of *Prevotella* was significantly higher in the low quality samples. Unsupervised clustering analysis demonstrated that the seminal bacterial communities were clustered into three main groups: *Lactobacillus*, *Pseudomonas*, and *Prevotella* predominant group. Remarkably, most normal samples (80.6%) were clustered in *Lactobacillus* predominant group. The analysis results showed seminal bacteria community types were highly associated with semen health. *Lactobacillus* might not only be a potential probiotic for semen quality maintenance, but also might be helpful in countering the negative influence of *Prevotella* and *Pseudomonas*. In this study, we investigated whole seminal bacterial communities and provided the most comprehensive analysis of the association between bacterial community and semen quality. The study significantly contributes to the current understanding of the etiology of male fertility.

## Introduction

### Semen quality and male infertility

Infertility is an increasingly common condition, and the male factors (either alone or in combination with female factors) are now estimated playing a significant role in about 40%–50% of infertile couples. Despite contemporary therapies undoubtedly raising the likelihood of conception among couples suffering from male infertility, these solutions often overlook the absence of a defined etiological or pathophysiological diagnosis. Male infertility, unfortunately, is still considered “idiopathic” in a large proportion of cases [1]–[3]. Consequently, there is a fundamental need to carry out research directed to establish the causes (and potential means of prevention) of male infertility.

Acute and chronic infections of the genitourinary (GU) tract may induce male factor infertility. Infectious etiologies cause about 15% of male factor infertility cases [4]. Ochsendorf [5] and Keck et al. [6] found that numerous infectious bacterial, viral, fungal, and protozoan species can enter the normal genital-urinary tract by route of sexual transmission, intracanicular spread of infected urine, or hematogenous seeding of genital organs. Infections of the testicle, epididymis, and prostate [7], [8] can negatively affect spermatogenesis and fertility. There are multiple causes of elevated seminal leukocytes (ESL) including infectious etiologies like genital-urinary infection and non-infectious etiologies including exposure to environmental toxins, man-made products during intercourse, tobacco products, alcohol and certain medications [9]. Other potent noninfection causes such as vasovasostomy, varicoceles, autoimmunity, defective spermatogenesis and poor sperm viability can lead to elevated seminal leukocytes [10], [11]. Bacteriospermia and the recruitment of seminal leukocytes can potentially impair male fertility through the deterioration of spermatogenesis, impairment of sperm function, and genital tract dysfunction and/or obstruction.

### Human microbiome and health

Microbiomes play an important role in human health, disease and some uncertain etiologies. Human skin, intestines, oral cavity, vagina and urethra can host microbial communities. The composition of microbiomes and their connection with various parts of the human body impact human health and the influence on the cause of disease [12]–[14]. The routine culturing methods and the polymerase chain reaction method are clinically useful for detecting specific aerobic, anaerobic or pathogenic bacteria in clinical specimens [15]. Next-generation sequencing technology can be used to directly extract large-scale microbial DNA and RNA sequences from human mixed microbial communities, and can also be used to sequence microbiomes which cannot be cultured [16]. Moreover, next-generation sequencing is more efficient than Sanger sequencing and less expensive [17]. 16S ribosomal RNA analysis is the most commonly-used approach to investigate the cultured-independent microbiomes. Microbiome identification can be achieved by sequencing 16S ribosomal RNA based on next-generation sequencing and bioinformatics approaches.

### Related works

Several bacteria identified in semen by previous studies have been associated with male infertility. Akutsu et al. performed PCR-based detection of 16S ribosomal RNA genes for six species and detected *Lactobacillus inner* and. *Gardnerella vaginalis* in 28% and 14% of semen samples, respectively [18]. Domes et al. determined the incidence of bacteriospermia and found elevated seminal leukocytes (ESL) in a subfertile male population. The rate of bacteriospermia was 15% using a concurrent culture of 22 species in 7,852 samples, and four most common bacterial species identified in seminal fluid were *E. fecalis* (56%), *E. coli* (16%), GBS (13%) and *S. aureus* (5%) [8]. Using a culture method for bacteria detection, Ibadin and Ibeh found 36 out of 87 semen samples of infertile men (41.4%) showed at least one pathogen. *Staphylococcus aureus* (16.9%), *Staphylococcus saprophyticus* (9.2%), *Escherichia coli* (6.9%), *Proteus mirabilis* (3.4%), *Klebsiella spp* (2.3%), *Pseudomonas aerouginosa* (1.1%) and *Proteus vulgaris* (2.3%) were identified as being associated with bacteriospermia and with the rate of total motility and morphologically abnormal sperms [19]. Manca et al. performed microscopic analyses and cultures on 696 semen specimens and found *Gardnerella vaginalis* was the most frequently isolated bacterium, followed by *Escherichia coli* and *Enterococcus sp*. In addition, sperm concentration, motility and morphology were most likely to deteriorate in the presence of *G. vaginalis* and *U. urealyticum* [20]. Moretti et al. performed semen culture and sperm transmission electron microscopy (TEM) analysis on 1,256 patients, with 417 samples (33.2%) showing the presence of bacterial species. The authors suggested that sperm bacterial contamination is quite frequent and could contribute to the deterioration of the sperm quality in infertile men [21]. Kiessling et al. performed PCR amplification of bacterial rDNA on 34 semen samples, and identified gram-positive anaerobic cocci, *Corynebacterium spp., Staphylococcus, Lactobacillus, Streptococcus spp., Pseudomonas spp., Haemophilus* and *Acinetobacter spp.* as the largest groups in different specimens. The authors found the concentration and motility of rDNA-positive specimens were not statistically different from the rDNA-negative specimens. In addition, the authors thought that the abundance of bacteria in semen is not commensal, arise from infection in the male genitourinary tract, may influence fertility, and may reflect an inadequate cellular immune response [22]. Hou et al. analyzed the microbiota of the seminal fluid from healthy and infertile men by using pyrosequencing V1–V2 regions of 16S rRNA gene [23]. The results showed that the bacterial community could be clustered in to six group, and no significant differences were found between sperm donors and the infertility patients. However, multiple statistical tests showed the presence of *Anaerococcus* has negative association with sperm quality.

**Figure 1:**
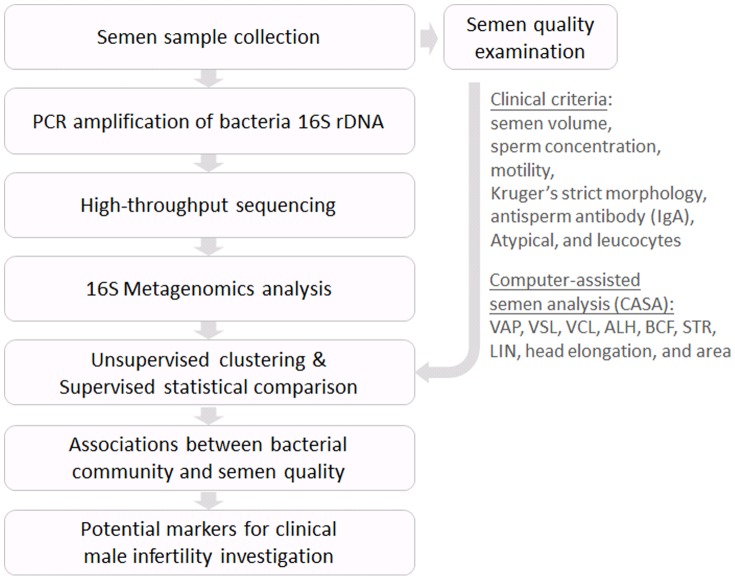
Research overview.

Bacterial identification methods performed in previous studies were either PCR-based or culture methods and, to date, a comprehensive understanding of bacterial communities in semen is still lacking. Most previous works only focused on a few types of bacteria and relied on qualitative analysis to discover associations between semen microbiomes and semen quality. We therefore present an analysis flow by combining next-generation sequencing technology and clinical semen quality examination (Fig. 1) to produce a high-resolution analysis of relationships between bacterial communities and semen quality.

## Results

As shown in Table 1, of the 96 men clinical examined for semen quality, 10 had abnormal semen volume; 13 had low semen concentration (<15×10^6^); 12 had low motility (<40%); 44 had low Kruger's morphology (<5%); 10 had abnormal antisperm antibodies (IgA) (>30%); 8 had atypical (≧1%) and 18 were found to have leucocytes. Table S1 summarizes participant metadata, including CASA values. Thirty-six semen samples without any abnormal clinical values were defined as the normal samples.

**Table 1:** Study participant characteristics and demographics.

Participant Characteristic	
Age	
Age range	26–58 years
Mean	35.75 years
Median	35 years
Semen volume	Number of samples
≧ 6.4 ml	4 (abnormal)
1.2–6.4 ml	86
≦1.2 ml	6 (abnormal)
Sperm concentration	Number of samples
≧15×10^6^	83
<15×10^6^	13 (abnormal)
Motility	Number of samples
≧40%	84
<40%	12 (abnormal)
Kruger's strict morphology	Number of samples
>14%	4
9–14%	18
5–9%	30
≦5%	44 (abnormal)
Antisperm antibody (IgA)	Number of samples
>30%	10 (abnormal)
≦30%	86
Atypical *	Number of samples
≧1%	8 (abnormal)
<1%	88
Leucocytes	Number of samples
Observed	18 (abnormal)
Unobserved	78

* Percentage of very small head of spermatozoa

**Figure 2:**
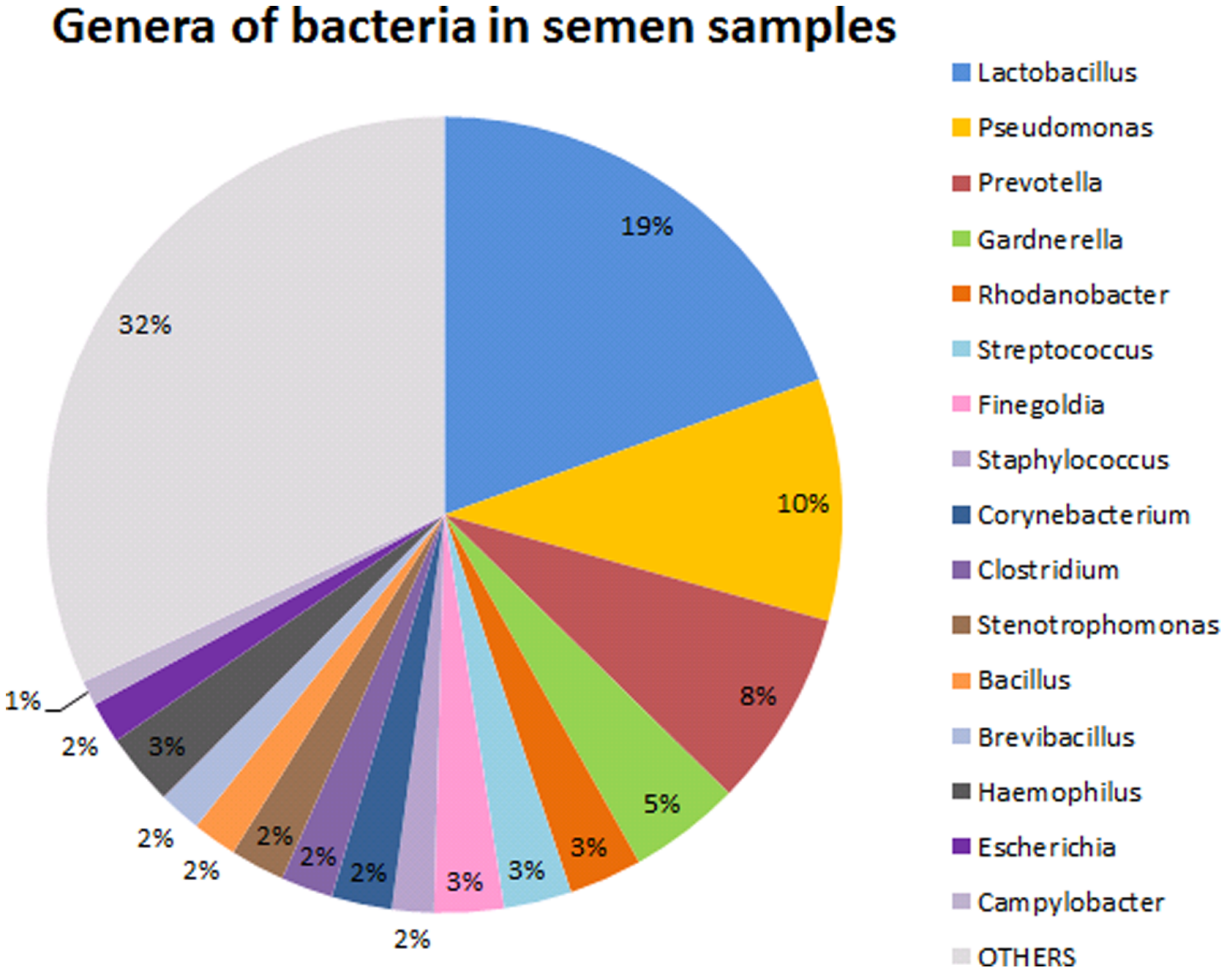
Relatively abundant genera in semen samples.

A total of 8,337,766 sequence reads was obtained from the 96 samples with a median read length of 125 bp and a mean of 80,424 reads per study participant. Sequence reads were passed through our taxonomic mapping flow and classified to represent seminal bacteria. An average number of 135 genera and 569 species were detected in the samples, and Table S2 summarizes the taxonomic assignment of sequence reads. In Schloss et al's study, the microbiome communities should be compared using an equal number of sequences to minimize the sequence artifact generated by high-throughput sequencing [24]. Therefore, we used the proportion instead of number of sequence reads to represent the taxonomic composition of each sample for further analysis. The distribution of relatively abundant genera is depicted in Fig. 2. The sequencing results showed that the most abundant species of bacteria in semen are *Lactobacillus iners* (14.09%), *uncultured Prevotella sp.* (3.06%), *uncultured Gardnerella sp.* (2.96%), *Lactobacillus sp.* (2.53%), *uncultured Pseudomonas sp.* (2.52%) and *Prevotella bivia* (2.22%) (Table S3). The most abundant genera of bacteria are *Lactobacillus* (19.9%), *Pseudomonas* (9.84%), *Prevotella* (8.51%), *Gardnerella* (4.21%), *Rhodanobacter* (2.74%), *Streptococcus* (2.74%), *Finegoldia* (2.73%) and *Haemophilus* (2.58%) (Table S4).

### Unsupervised Clustering Analysis

**Figure 3:**
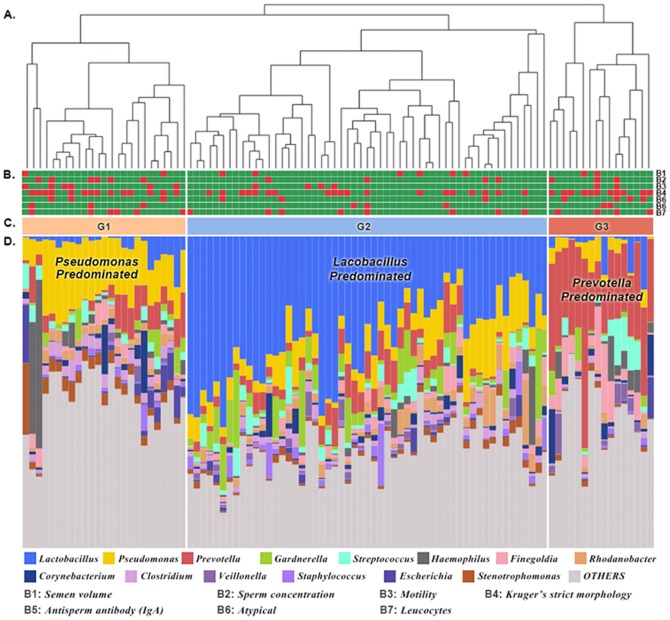
Bacterial communities in semen samples. (A) Hierarchical clustering was used to generate a clustering tree depicting the bacterial diversity in 96 men with clinical values. The scale bar represents the sample distance generated by UniFrac. (B) Clinical value status is depicted in the seven horizontal bars. The red and green rectangles respectively represent abnormal and normal clinical values. B1 to B7 respectively represent the clinical value status of semen volume, sperm concentration, motility, Kruger's strict morphology, antisperm antibody (IgA), Atypical, and leucocytes. (C) G1, G2 and G3 represent the three main groups in the clustering results, which were respectively predominated by *Pseudomonas*, *Lactobacillus* and *Prevotella*. (D) The colored bars represent the taxonomic compositions in each sample. Less abundant taxa were grouped in the “Others” category.

**Figure 4:**
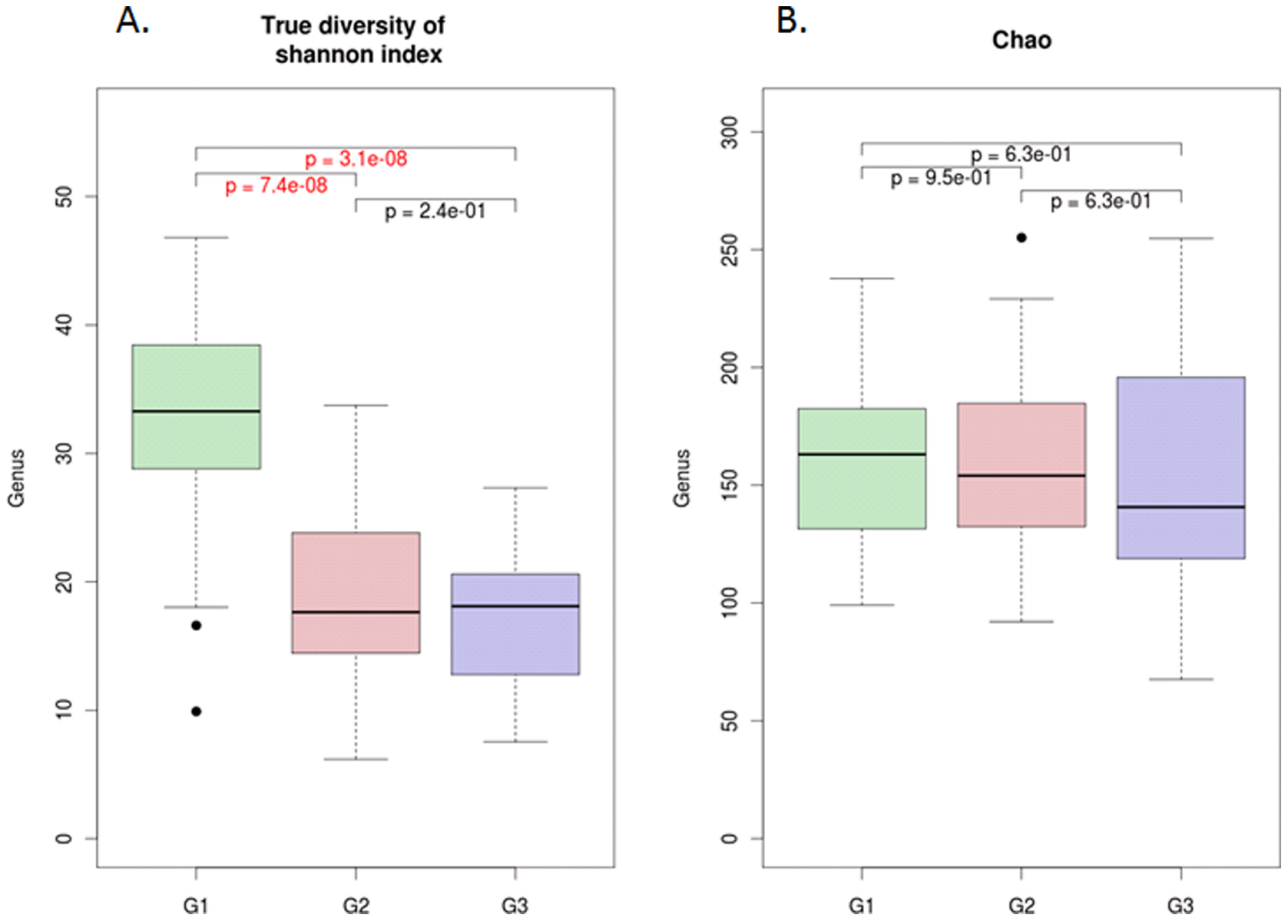
Diversity and richness of bacterial communities in the G1, G2 and G3 groups.

A hierarchical clustering was performed, and the seminal bacterial community and clinical values of each sample are shown in Fig. 3. The results demonstrate that the bacterial communities in semen are clustered into three main groups, G1, G2 and G3 (Fig. 3*C*). Five out of 25 samples (20%) in *Pseudomonas*-predominant group (G1), 2 out of 16 samples (12.5%) in *Prevotella*-predominant group (G3), and 29 out of 55 samples (52.7%) in *Lactobacillus*-predominant group (G2) are normal samples, and both of G1 and G2 are statistically different from G2 (fisher p-value = 7.38E-03 and 4.48E-03). Figure 3 shows that the most abundant genera in G1, G2, and G3 were *Pseudomonas* (16.1%), *Lactobacillus* (32.3%), *and Prevotella* (26.3%), respectively. Remarkably, 29 out of 36 normal samples (80.6%) were clustered in the *Lactobacillus*-predominant group (G2). The results showed that seminal bacteria community types were highly associated with semen health. The genera diversity and richness analysis showed that the G1 group exhibited significantly higher levels of bacterial diversity than G2 and G3 group (Fig. 4*A*), and there were no significant difference of richness among three groups (Fig. 4*B*).

**Figure 5:**
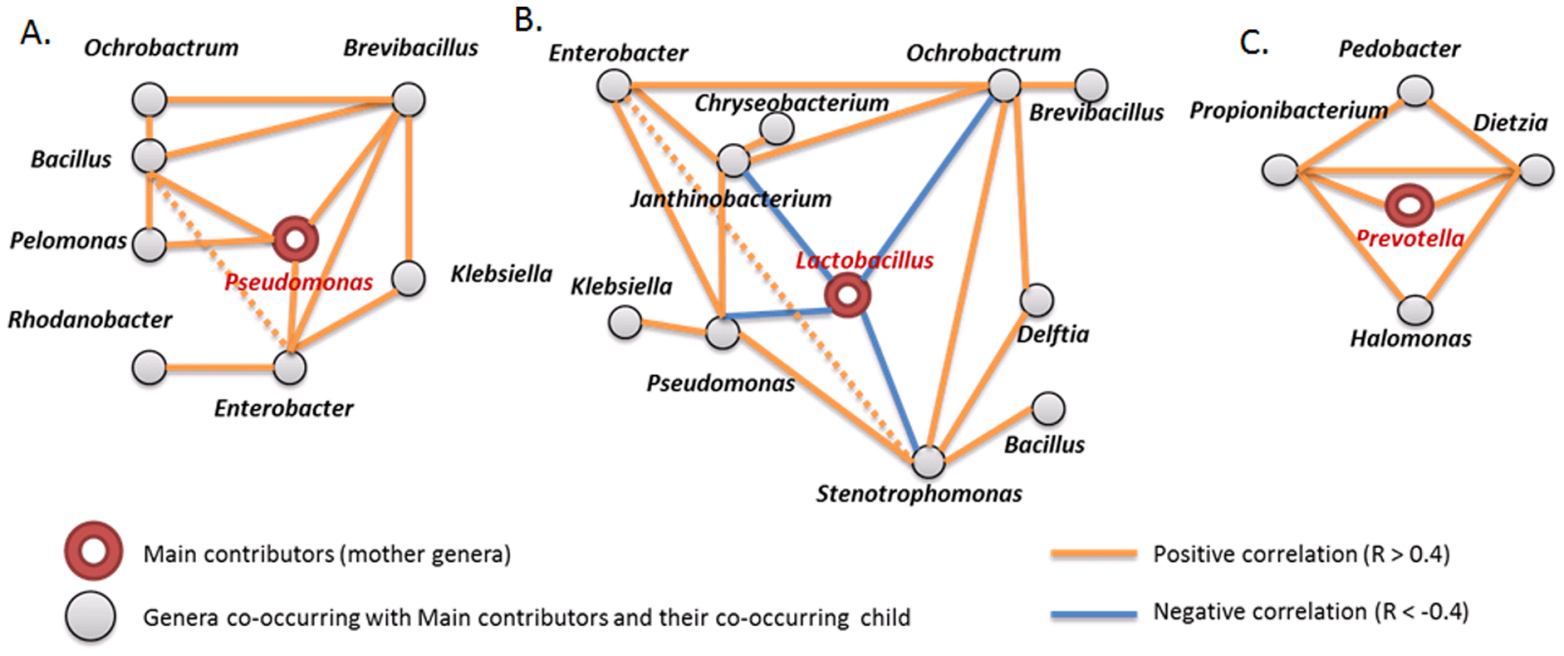
Genera co-occurring with main contributors and their co-occurring children in the (A) G1, (B) G2 and (C) G3 groups.

To determine the functional basis of the microbiome community types (G1, G2 and G3), we investigated differences in their composition and correlation to abundance of co-occurring genera (Fig. 5). Semen microbiome community type G1 (with 25 samples) is enriched in *Pseudomonas*, which co-occurs, for example, with *Enterobacter*. Both *Pseudomonas* and *Enterobacter* are Gram-negative bacteria and several strains of these bacteria are pathogenic and cause opportunistic infections in immunocompromised hosts, most commonly in the urinary tract. Microbiome community type G2 is the most frequent, containing 55 samples, and is enriched in *Lactobacillus*, which is Gram-positive facultative anaerobic, and negatively correlated to *Pseudomonas, Stenotrophomonas, Ochrobactrum* and Janthinobacterium, which all are Gram-negative bacteria. *Lactobacilli* have been found to play an important role of restoring particular physiological balances such as in the vaginal microbiome [25]–[27]. Microbiome community type G3 contains 16 samples and is enriched in *Prevotella* which mainly co-occurs with *Propionibacterium* and *Dietzia*. *Prevotella spp*. is a type of oral and vaginal flora and has been isolated from abscesses and burns in the vicinity of the mouth, urinary tract infections, and bacteremia associated with upper respiratory tract infections.

### Supervised comparison of seminal bacterial communities

**Figure 6:**
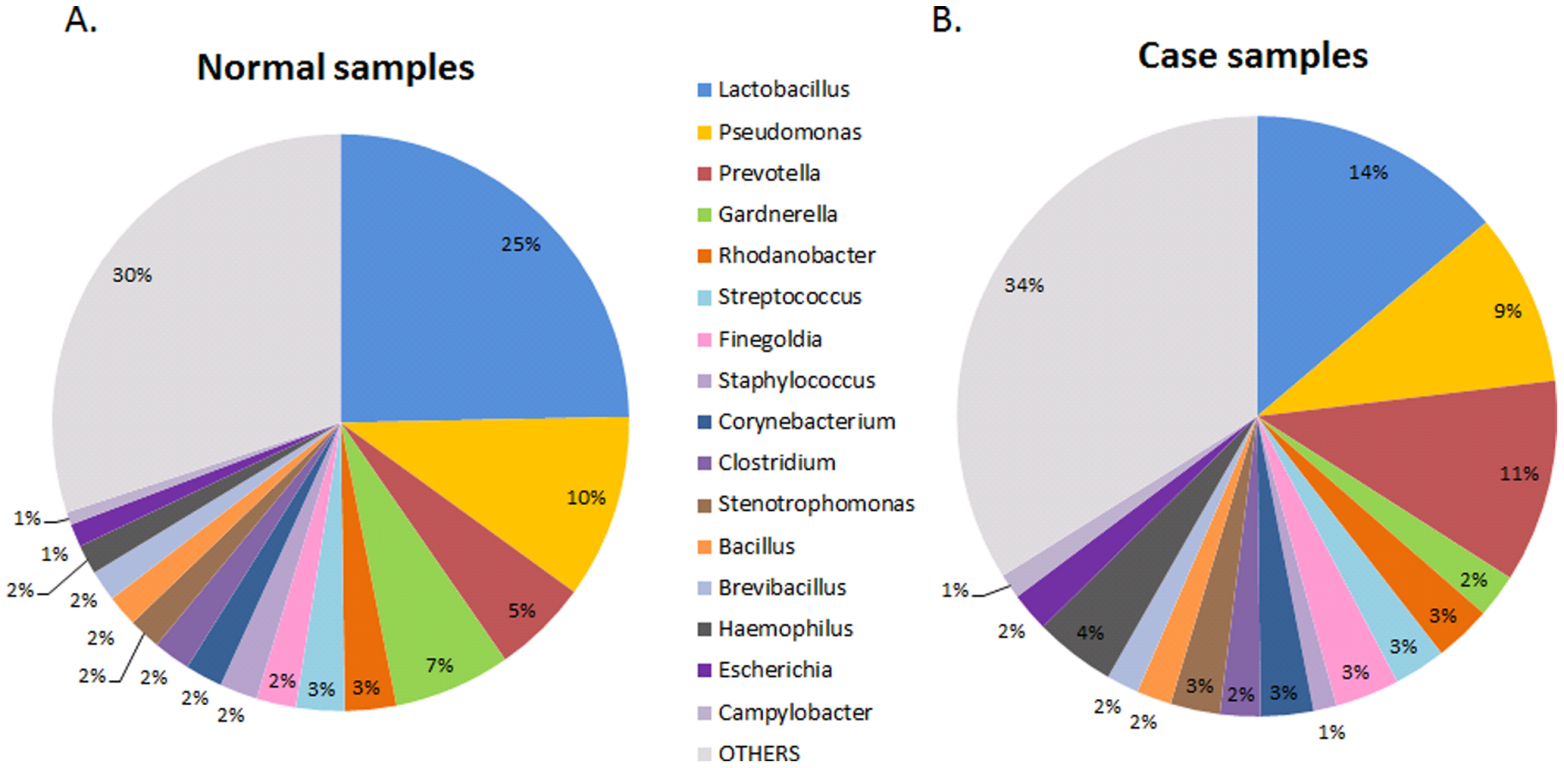
Relatively abundant genera in the (A) normal and (B) case samples.

**Figure 7:**
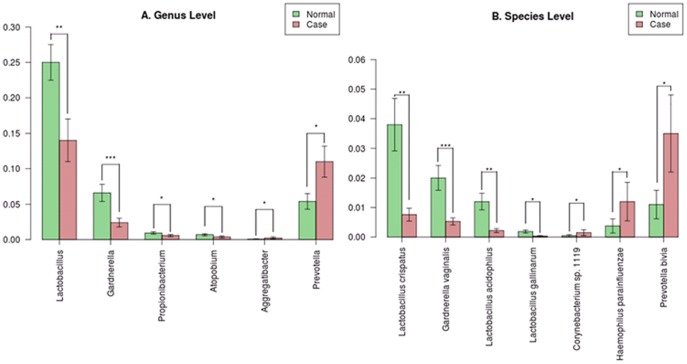
Relatively abundant bacteria with significantly different distributions between the normal and case samples (* p<0.05, ** p<0.01, *** p<0.001).

**Figure 8:**
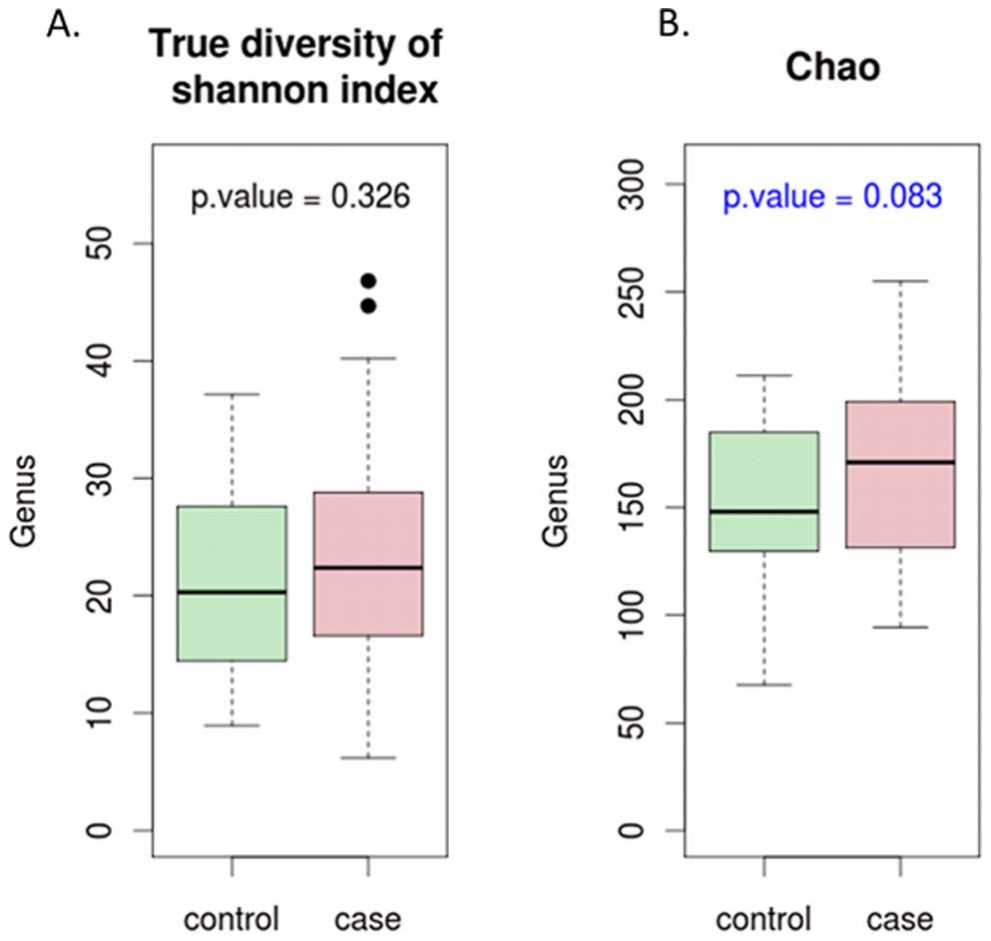
Diversity and richness of bacterial communities in the case and normal samples.

To investigate the association between bacterial community and semen quality, 36 semen samples examined without any abnormal clinical values were defined as normal samples, and 33 samples with more than two abnormal clinical values were used as case samples which were low-quality and more likely to be associated with male infertility. Figure 6 shows that the most abundant genera of bacteria in the normal samples were *Lactobacillus* (24.7%), *Pseudomonas* (10.3%), *Gardnerella* (6.6%), *Prevotella* (5.4%), *Rhodanobacter* (2.9%) and *Streptococcus* (2.7%), and the most abundant genera of bacteria in the case samples were *Lactobacillus* (13.8%), *Prevotella* (11%), *Pseudomonas* (9.3%), *Haemophilus* (4.4%), *Finegoldia* (3.5%), *Rhodanobacter* (3.1%), *Corynebacterium* (2.8%) and *Streptococcus* (2.8%). The normal samples had a significantly higher proportion of *Lactobacillus, Gardnerella, Propionibacterium* and *Atopobium*, while the case samples had a significantly higher proportion of *Prevotella* and *Aggregatibacter* (Fig. 7*A*). *Lactobacillus crispatus, Gardnerella vaginalis, Lactobacillus acidophilus* had a higher proportion in the normal samples, while *Prevotella bivia* and *Haemophilus parainfluenzae* had a higher proportion in the case samples (Fig. 7*B*). *Haemophilus parainfluenzae* is as an opportunistic pathogen which causes systemic diseases including endocarditis, meningitis and bacteremia, and is often isolated from the sputa of patients with chronic obstructive lung disease. The genera diversity and richness analysis showed that there were no significant differences in diversity between the case and normal samples (Fig. 8*A*), and the bacterial richness of the case samples was significantly higher than that of normal samples (Fig. 8*B*).

*Lactobacillus* and *Prevotella* not only constituted the major proportion of seminal microbiota in the normal samples and case samples, but also had significantly different proportions between the case and normal samples. Therefore they may respectively have strong positive and negative impacts on semen quality. *Pseudomonas* was a common genus in both case samples (10%) and normal samples (9%), and some species were considered to be opportunistic human pathogens. The proportion of *Pseudomonas* was not significantly different between the case and normal samples, and therefore *Pseudomonas* seems to have no impact on semen quality. However, further analysis results (Table S5) showed that *Pseudomonas* can contribute to the deterioration of semen quality in samples containing less *Lactobacillus* (Fisher's exact p-value = 0.04), such as the samples in the microbiome type G2 (*Pseudomonas*-predominated) group. *Lactobacillus* might not only be a potential probiotic for maintaining semen quality, but also might protect against the negative influence of *Prevotella, Haemophilus* and *Pseudomonas*.

**Figure 9:**
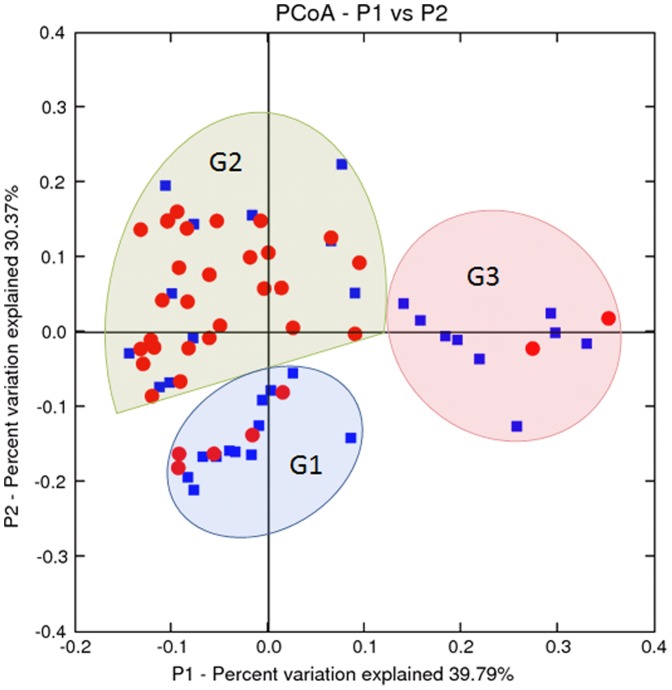
PCoA analysis of case and normal samples. The blue rectangles and red circles respectively represent the case and normal samples.

A principal coordinates analysis (PCoA) was performed to visualize seminal bacterial communities for the case and normal samples. As shown in Fig. 9, most normal samples (red circle) were located in the top left area, and most case samples (blue rectangle) were located on the bottom and right side. The nodes in the PCoA graph could be separated into three clusters according to the distribution of the red circles and blue rectangles. Interestingly, these three clusters also correspond to the G1, G2 and G3 groups as previously defined. Table S6 shows that the G1 and G3 groups respectively have a 5.2-fold and 8.5-fold greater likelihood of low semen quality than samples in the G2 group, which indicates that semen quality is highly associated with seminal bacterial communities.

Tables S7∼S10 respectively list the genera and species of bacteria significantly abundant in the samples with normal or abnormal clinical values. The genera and species which were significantly associated more than two clinical criteria are respectively summarized in Tables 2 and 3. The presence of *Lactobacillus* and *Gardnerella* respectively exert a positive association with five and six clinical criteria, while the presence of *Prevotella* and *Bordetella* respectively exert a negative association with three and four clinical criteria. In addition, only one bacteria genus (*Shewanella*) and one species (*Fusobacterium periodonticum*) were found to be significantly associated with semen volume (Table S8 and Table S10), which indicates that semen volume may be mainly caused by other etiological factors.

**Table 2:** Genera of bacteria significantly associated with more than 2 clinical criteria.

	Proportion.median	Proportion mean	Semen volume	Sperm concentration	Motility	Kruger's strict morphology	Antisperm antibody (IgA)	Atypical	Leucocytes
*Prevotella*	3.34%	5.36%				xx		xx	x
*Haemophilus*	0.20%	1.66%						x	x
*Campylobacter*	0.17%	0.72%				x		xx	x
*Peptoniphilus*	0.12%	0.45%				x		x	
*Brevibacterium*	0.02%	0.20%		x					x
*Pasteurella*	0.06%	0.17%		x					x
*Sphingobium*	<0.01%	0.09%		xx			x		x
*Dermacoccus*	<0.01%	0.09%		x					xx
*Aggregatibacter*	<0.01%	0.07%		x				x	x
*Varibaculum*	<0.01%	0.04%				x		xx	
*Lactobacillus*	25.42%	24.68%		++		++		+	++
*Gardnerella*	3.83%	6.56%		+++	++	+++	+	+	++
*Atopobium*	0.33%	0.70%		+					+

+, ++ and +++ indicate that the genus is significantly abundant in samples with normal clinical value under a Mann–Whitney U-test p value of 0.05, 0.01 and 0.001, respectively.

x, xx and xxx indicate that the genus is significantly abundant in samples with abnormal clinical value under a Mann–Whitney U test p value of 0.05, 0.01 and 0.001, respectively.

**Table 3:** Species of bacteria significantly associated with more than 2 clinical criteria.

	Proportion median	Proportion mean	Semen volume	Sperm concentration	Motility	Kruger's strict morphology	Antisperm antibody (IgA)	Atypical	Leucocytes
Arthrobacter sp. Zn12	<0.01%	0.15%		x	x		x		
Sphingobium estrogenivorans	<0.01%	0.07%		xx			x		
Varibaculum cambriense	<0.01%	0.04%				x		xx	
Prevotella sp. BV3C7	<0.01%	0.03%				x		x	
Actinomyces sp. 'Smarlab BioMol-2300463'	<0.01%	0.03%						x	x
Porphyromonas somerae	<0.01%	<0.01%		xx	x				
uncultured Gardnerella sp.	2.98%	4.53%		+++				+	
Lactobacillus crispatus	1.43%	3.76%		++		++	+		++
Gardnerella vaginalis	0.87%	2.03%		+++	++		++	+	+++
Lactobacillus acidophilus	0.37%	1.17%		+		+			++
Atopobium vaginae	0.29%	0.56%		++			+		
Pseudomonas sp. ps10-13	0.07%	0.10%		++			++	+	

+, ++ and +++ indicate that the specie is significantly abundant in samples with normal clinical value under a Mann–Whitney U-test p value of 0.05, 0.01 and 0.001, respectively.

x, xx and xxx indicate that the specie is significantly abundant resent in samples with abnormal clinical value under a Mann–Whitney U test p value of 0.05, 0.01 and 0.001, respectively.

The genera and species of bacteria associated with CASA criteria are respectively summarized in Table S11 and Table S12. Only morphometric criteria of CASA, head elongation and area were found to be associated with bacteria. Noticeably, *Lactobacillus crispatus* was not only associated with sperm elongation, but was also associated with Kruger's strict morphology (Table S9), which indicates that it may have a significant influence on semen morphology.

### Potential markers for classification of semen microbiome communities

**Figure 10:**
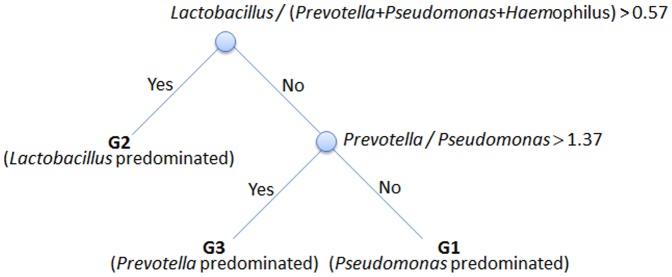
Classification rule and potential markers for classifying microbiome communities of semen.

Analysis results demonstrate that microbiome communities are significantly associated with semen quality (Table S6). PCR-based detection can be used to determine the critical bacteria for classification of particular semen microbiome communities in practical clinical investigations, and such results might be helpful for indicating possible causes of male infertility. Here, five abundant genera of bacteria, *Lactobacillus*, *Gardnerella*, *Prevotella*, *Pseudomonas*, and *Haemophilus*, were collected to classify 96 samples into three different microbiome community types using the machine learning method J48 in Weka 3.6.7 [28]. The proportion of each bacteria and the relative ratio between the different bacteria types were taken into consideration as features for rule-based clustering. Five-fold cross-validation was used to evaluate the performance of the classification model. As shown in Table S13, the classifier performed well in all three groups, and the ROC area of G1, G2 and G3 achieved 0.929, 0.953 and 0.947, respectively. As shown in Fig. 10, the classification rules are simple and are described as follows: (1) If the ratio of *Lactobacillus*/(*Prevotella*+*Pseudomonas*+*Haemophilus*) of a sample is greater than 0.57, the sample is classified to G2. (2) For a sample with a ratio of *Lactobacillus*/(*Prevotella*+*Pseudomonas*+*Haemophilus*) below 0.57, it is classified to G3 if the ratio of *Prevotella*/*Pseudomonas* is greater than 1.37; otherwise, it is classified to G1. The results show that *Lactobacillus*, *Prevotella*, *Pseudomonas*, and *Haemophilus* could potentially be markers for future clinical applications and investigations of male infertility.

## Discussion

This study reveals whole bacterial communities in semen and has potentially high clinical value of the high-throughput sequencing of bacterial 16S rRNA in semen specimens to help monitor semen quality. Three seminal microbiome community types were identified: G1 (*Pseudomonas*-predominant group), G2 (*Lactobacillus*-predominant group) and G3 (*Prevotella*-predominant group) (Fig. 3). Twenty-nine out of 36 normal samples (80.6%) were clustered in the G2 group, which accounts for more than half of the samples in G2 (52.7%). Only 5 out of 25 samples (20%) in G1 group and 2 out of 16 samples (12.5%) in G3 group were normal samples. In addition, comparative analysis shows that samples in the G1 and G3 groups respectively have a 5.2-fold and 8.5-fold greater chance than the G2 group of containing more than two abnormal clinical values. These results indicate that semen quality is highly associated with seminal bacterial communities.

As compared to Hou et al.'s study [23], the different results were discovered in our study. It might be caused by use of different samples, clinical examinations, sperm quality groups and clustering methods between two studies. However, most abundant genera present in Hou et al's study were also identified abundant in our data, such as *Lactobacillus* (19.9%), *Prevotella* (8.51%), *Finegoldia* (2.73%), *Corynebacterium* (2.04%), *Staphylococcus* (1.46%) and *Veillonella* (1.04%). In addition, some of our relative abundant bacteria had been identified in previous studies which used PCR-based or culture methods to detect bacteria in semen or the male genital tract, such as *Lactobacillus* [18], [22], [29], *Pseudomonas* [22], [29], *Gardnerella* [18], [20], [30], [31], *Prevotella* [29], [32] and *Haemophilus* [22]. It shows the comparability and reliability of our sequencing data.

As shown in Fig. 7*A*, genera of *Lactobacillus*, *Gardnerella*, *Propionibacterium* and *Atopobium* were relatively abundant and significantly present in the normal samples, and *Prevotella* and Aggregatibacter were relative abundant and significantly present in the case samples. *Lactobacillus* is a genus of Gram-positive anaerobic bacteria [33], and is a major part of the lactic acid bacteria group. *Lactobacillus* was detected in 95 of the 96 samples (98.96%), with proportions ranging from 0% to 61.22% with an average of 19.90%. In humans, *Lactobacilli* are present in the vagina [34] and the gastrointestinal tract. *Lactobacilli* can also be used to restore physiological balance such as in the vaginal eco-system [25]–[27]. In previous studies, *Lactobacillus* had been reported as a normal bacteria in semen [35], [36], and is found to have a positive impact on clinical criteria for leucocytes, Kruger's strict morphology, sperm concentration, and Atypical. *Gardnerella* is a genus of Gram-variable-staining anaerobic bacteria [37], [38] of which *Gardnerella vaginalis* is the only species. *Gardnerella* was detected in 93 of the 96 samples (96.88%) in proportions ranging from 0% to 28.82% with an average of 4.21%. *G. vaginalis* is the predominant cause of bacterial vaginosis in women by disrupting normal vaginal microflora [39]. *Propionibacterium* is a genus of Gram-positive bacteria. Its members are primarily facultative parasites and commensals of humans. *Atopobium* is a genus of anaerobic Gram-positive bacteria. In Akutsu et al's study, 16S ribosomal RNA genes of *Lactobacillus crispatus* and *Atopobium vaginae* were detected in vaginal fluid and female urine samples [18]. *Prevotella* is a genus of Gram-negative anaerobic bacteria [40], and it had been reported to be involved in bacterial vaginosis [41], [42]. *Aggregatibacter* is a genus of Gram-negative bacteria. *Pseudomonas* is a genus of Gram-negative aerobic bacteria [43]. *Pseudomonas* has been known to cause bloodstream infections, and even to be associated with bacteraemia [44], [45]. *Pseudomonas aeruginosa* is the type species of the genus [46], and it is increasingly recognized as an opportunistic pathogen of clinical relevance. *Pseudomonas aeruginosa* is one type of bacteria detected in the semen of infertile men [47]. *Pseudomonas putida* is a bacterium commonly found in genitourinary tract infections [48]–[50].

As shown in Fig. 6, *Pseudomonas* was a common genus in both case samples (10%) and normal samples (9%). The proportion of *Pseudomonas* dose not significantly differ between the case and normal samples, and thus seemingly has no association with semen quality. As seen in Table S5, *Pseudomonas* was found to contribute to the deterioration of semen quality in samples containing less *Lactobacillus*. According to the bacteria discovered in Fig. 7*A* and the above discussion, it indicated that Gram-positive bacteria, such as *Lactobacillus, Propionibacterium* and *Atopobium*, seem to be involved in not only facilitating semen quality maintenance, but also might protect against the negative influence of Gram-negative bacteria, such as *Prevotella*, *Aggregatibacter* and *Pseudomonas*. In our opinion, *Lactobacillus* supplements could help maintain semen quality and increase fertility potential, and future studies are recommended to validate this assumption.

Previous studies have used PCR-based or culture methods to detect *Gardnerella vaginalis* in semen or the male genital tract [18], [20], [30], [31]. *G. vaginalis* is considered to be a pathogen in the lower male genital tract [20], [51], [52]. Manca *et al.* found a positive correlation between the presence of *G. vaginalis* infections and leukocytes, while sperm concentration, motility and morphology were found to deteriorate in the presence of *G. vaginalis* [20]. Andrade-Rocha concluded that the presence of *G. vaginalis* is not associated with either abnormal sperm characteristics or inflammatory response in infected men [30]. However, in this study, we found a positive correlation between the proportion of *G. vaginalis* genera and semen quality (see Table 3), including sperm concentration, motility, Kruger's strict morphology, antisperm-antibody (IgA), Atypical and Leucocytes – findings which differ from previous studies. In our data, *G. vaginalis* was relatively abundant in the G2 group which is predominated by *Lactobacillus*. We hypothesize that *G. vaginalis* might act as an opportunistic microorganism which can disturb the bacterial ecosystem to shift from commensal to pathogenic bacterium in low quality semen. Therefore, the positive association between *G. vaginalis* and semen quality might be the accompanied effect provided by *Lactobacillus*. Nevertheless, the role of *G. vaginalis* remains unclear since it is found in both normal and abnormal fertile individuals [53].

The presence of *Lactobacillus crispatus* was found to have a positive association with quality of sperm concentration, leucocytes, IgA, and Kruger's strict morphology (Table S9). It was also found to be related to sperm elongation in CASA criteria (Table S12). *Lactobacillus crispatus* is a normal vaginal flora [54] but its significant to semen is unknown [22]. In healthy women, *Lactobacillus crispatus* or *Lactobacillus iners* are the dominant vaginal bacterial communities [55]. Also, in co-culture conditions, *Lactobacillus crispatus* reduced the viability of *Gardnerella vaginalis* and *Prevotella bivia* [56], and *Lactobacillus crispatus* was found to restore normal microbial communities in the vaginal ecosystem by displacing vaginal pathogens [57]. Our analysis results indicate that *Lactobacillus crispatus* has the potentialin maintaining the semen ecosystem and semen quality, as the role in the vaginal ecosystem.

*Escherichia coli* was found to be associated with reduced semen density and diminished progressive motility [58], and could cause a decrease in *in vitro* viability [59] and motility [59]–[61]. In De Francesco et al's study, *E. coli* was the second most frequently isolated bacterium in 696 infertile men, and its concentration differed significantly between infertile and fertile males [20]. However, it was not detected using the PCR-based method by either Jarvi [62] or Kiessling [22], and was detected in only 3 of 17 specimens by Tanner [63]. Eyre et al. suggested that, although *E. coli* may be present in semen, the number of organisms are too few to compete in PCR reactions, but that they are more readily cultured than other organisms such as gram-positive anaerobic cocci (GPAC). In this study, we clearly identified the proportion of *E. Coli* in each sample using high-throughput sequencing technology. *Escherichia coli* were detected in 94 of the 96 samples (97.92%) in proportions ranging from 0% to 16.42%, with an average of 1.26%. Analysis results showed that *E. Coli* were not associated with semen quality (p>0.05 for the 7 detected clinical criteria, data not shown).

Little is known about the composition of the microflora in normal seminal fluid or the male genital tract [23], [36]. As far as we know presently, the microflora in semen of healthy men was characterized by Gram-positive bacteria, e.g. lactobacilli, coagulase-negative staphylococci, streptococci and corynebacteria, [36], [64] by culture detection.

Recently, the composition of microflora in semen of healthy men detecting by next generation sequencing has been reported in the work of Hou et al. [23]. Corynebacterium (14%) was the most abundant genus in healthy men, followed by Ralstonia (13%), Lactobacillus (9%), Streptococcus (7%), Finegoldia (7%), and Anaerococcus (5%). These genera were also isolated in infertility patients and did not show significant differences between healthy men and patients.

In this study, microbiome type G2 (*Lactobacillus* predominated) was found to be associated with healthy semen, while G1 (*Pseudomonas* predominated) and G3 (*Prevotella* predominated) were found to be associated with low quality semen. However, the G2 group accounts for 23.6% of the case samples, which indicates that some instances of low quality semen may be caused by genetic or other etiological factors. In addition, the G1 and G3 groups respectively contained 20% and 12.5% normal samples, which largely corresponds to the results of previous studies [21], i.e., the bacteria contamination of semen samples of fertile individuals did not compromise sperm quality, but it is possible that the presence of bacteria further deteriorated the overall quality of seminal plasma in infertile men.

Finally, a practical criterion was developed to classify semen samples into three different microbiome community types by using four genera of bacteria: *Lactobacillus*, *Prevotella*, *Pseudomonas*, and *Haemophilus*. The fertility status and the fertility outcome of our samples are not available currently. A case-control study will be conducted in the near future to clarify this issue and validate our classification model. Although the mechanism by which pathogenic bacteria cause infertility is still unknown, these four genera could serve as potential markers for future clinical applications and investigations of male infertility.

## Materials and Methods

### Ethics Statement

The study was approved by the institutional review board of Mackay Memorial Hospital (Hsinchu, Taiwan). All patients provided written informed consent.

### Sample collection

Ninety-six semen samples were collected from 96 patients at the reproductive center at Mackay Memorial Hospital (Hsinchu, Taiwan). The patients were couples suffering from either male infertility or female infertility, or infertility of unknown etiologies. The patients had been suffering from primary infertility for at least one year, and did not suffer from other significant health issues. Semen was collected by masturbation into a sterile bottle following 3–5 days of sexual abstinence. All semen samples were collected using the Copan ESwab collection Kit 480C into Eswab tubes for transport, and stored at 4°C for DNA extraction and PCR amplification of bacteria 16S rDNA.

### Measurement of semen quality

All semen samples were allowed to liquefy for 30 minutes at 37°C, followed by assessment of sperm parameters according to World Health Organization (WHO) guidelines [65]. Leukocytes were identified by peroxidase stain. Leukocyte concentrations below 1×10^6^ cells/ml are considered normal under the WHO guidelines.

Sperm concentration and motion parameter analysis were assessed using the HTM-IVOS semen analyzer (Hamilton Thorne Research, Beverly, MA, USA) and manually monitored. Sperm motion was examined after mixing the sperm suspension and loading a 5 µl aliquot into a chamber of the 20-µl M-4 chamber MicroCell slide (Conception Technologies, La Jolla, CA, USA). The slide was then transferred to the HTM-IVOS semen analyzer where it was maintained at 37°C for 2 minutes before data assessment, which was conducted on randomly selected fields. Within each sample, computer-assisted semen analysis (CASA) was performed to measure 9 aspects of semen quality, including average path velocity (VAP), straight-line (progressive) velocity (VSL), curvilinear velocity (track speed) (VCL), lateral amplitude (ALH), beat frequency (BCF), straightness (STR), linearity (LIN), head elongation, and area.

Sperm antibody tests, including IgA classes, were conducted using SpermMar Test (Fertipro N.V., Belgium) by two well-experienced technicians. Sperm morphology was assessed using Kruger's strict criteria [14] after slide staining with Diff-Quik (Diagnostics AG, Medion, Switzerland) by two other well-experienced technicians to classify head defects, neck and midpiece defects and tail defects of the spermatozoa. Very small spermatozoa heads were identified as an atypical morphology indicating immaturity issues.

### DNA extraction

DNA extraction was performed directly on the samples using a QIAamp DNA Blood Mini Kit (Qiagen). Each sample was transferred to a 1.5 ml microcentrifuge tube, and centrifuged at 13,000 rpm for 2 min to pellet the bacteria. Bacterial pellets were suspended in 180 µl of the appropriate enzyme solution, and incubated for at least 30 min at 37°C. In addition, 20 µl proteinase K and 200 µl Buffer AL was added to the sample, and mixed by vortexing. Each suspension was incubated at 56°C for 30 min and then for a further 15 min at 95°C. The 1.5 ml microcentrifuge tube was briefly centrifuged to spin down the suspension. From this point, the extraction proceeded following the protocol of the QIAamp DNA Blood Mini Kit. The DNA was eluted with 30 µl Buffer AE, and centrifuged at 8,000 rpm for 1 min. The DNA extract was stored at −20°C for further analysis.

### Library construction

Two PCR primers, F515 (5′-GTGCCAGCMGCCGCGGTAA-3′) and R806 (5′-GGACTACHVGGGTWTCTAAT-3′), were designed to target the V4 domain of bacterial 16S rRNA as described in a previous study [66]. The PCR amplification was performed in a 50 µl reaction volume containing 25 µl 2X Phusion Flash Master Mix (ThermoFisher), 0.5 µM of each forward and reverse primer, and 50 ng DNA template. The reaction conditions consisted of an initial 98°C for 30 sec followed by 30 cycles of 98°C for 10 sec, 54°C for 30 sec, 72°C for 30 sec, and a final extension of 72°C for 5 min. Amplified products were checked by 2% agarose gel electrophoresis and ethidium bromide staining. Amplicons were purified using the AMPure XP PCR Purification Kit (Agencourt), and quantified using a Qubit dsDNA HS Assay Kit (Qubit) on a Qubit 2.0 Fluorometer (Qubit) all according to the respective manufacturers' instructions. For V4 library preparation, Illumina adapters were attached to the amplicons using the TruSeq DNA Sample Preparation v2 Kit (Illumina). Purified libraries were applied for cluster generation and sequencing on the Miseq system.

### 16s rRNA sequence data quality filtering and taxonomy mapping

Paired-end sequences were obtained using an illumina sequencing machine in FASTQ format and sequence quality was assessed using the FASTX-Toolkit. The raw reads were pre-processed to classify samples by barcodes, trim barcodes and truncate low quality bases and reads. The quality of reads exceeding a Phred quality score 30 (Q30) were retained. Sequences consisting of fewer than 100 nucleotides were discarded along with any reads containing ambiguous characters. A fast and memory-efficient aligning sequencing reading tool [67] was adopted to map the paired-end reads to bacterial 16s ribosomal RNA (rDNA) sequences obtained from NCBI 16S ribosomal RNA sequence database and NCBI nucleotide collection database. The reads were mapped to specific bacteria if the sequence similarity exceeded 97% and paired-end reads were aligned to the same reference sequence. The raw sequencing data are available at http://clinic.mbc.nctu.edu.tw/semen/.

### Bacterial community analysis

An unsupervised clustering method was applied to observe relationships between the samples and to explore taxonomic associations with semen quality. The Greengenes core set tree was used to represent the distance between bacteria [68], [69], and the phylogeny-based weighted UniFrac [70] was applied to calculate the difference in overall microbial community composition. The pseudo F statistic developed by Calinski and Harabasz [71] was applied to generate the optimum number of clusters. A dendrogram was constructed using MEGA 4.0 [72]. Statistics for true diversity through the Shannon index and Chao richness were calculated separately for each sample using R language. Correlations of co-occurring network were computed on the basis of pairwise Spearman correlation analysis. The correlations with an absolute Spearman correlation above 0.4 were transformed to links between two genera in the genus network. The Mann–Whitney U test was used to investigate significant genera or species between case and normal samples. The bacteria with proportion more than 0.25% were collected for multiple testing with false discovery rate less than 0.05 by using adaptive Benjamini-Hochberg method [73]. UniFrac distance matrices which represent the characteristics between microbial communities of samples dependent on phylogenetic information was transformed into principal coordinated using Principal coordinates analysis (PCoA) [74] to provide a visualization of the sample distribution patterns.

## Supporting Information

Table S1 Participant metadata.
(XLS)

Table S2 Taxonomic assignment of sequence reads.
(XLS)

Table S3 Identified species of bacteria in semen.
(XLS)

Table S4 Identified genera of bacteria in semen.
(XLS)

Table S5 The association between *Pseudomonas* and semen quality in samples with and without relatively abundant *Lactobacillus*.
(DOCX)

Table S6 Fisher's exact test and odds ratio of low semen quality rates comparing G1 and G3 to G2.
(DOCX)

Table S7 Genera of bacteria significantly abundant in samples with normal clinical value.
(DOCX)

Table S8 Genera of bacteria significantly abundant in samples with abnormal clinical value.
(DOCX)

Table S9 Species of bacteria significantly abundant in samples with normal clinical value.
(DOCX)

Table S10 Species of bacteria significantly abundant in samples with abnormal clinical value.
(DOCX)

Table S11 Genera of bacteria significantly associated with CASA criteria.
(DOCX)

Table S12 Species of bacteria significantly associated with CASA criteria.
(DOCX)

Table S13 Performance classification of microbiome community types using four bacteria, *Lactobacillus*, *Prevotella*, *Pseudomonas* and *Haemophilus*.
(DOCX)
