# Supplementary material for: Bacterial Communities in Semen from Men of Infertile Couples: Metagenomic Sequencing Reveals Relationships of Seminal Microbiota to Semen Quality
Source: PLoS One. 2014 Oct 23;9(10):e110152. doi: 10.1371/journal.pone.0110152 (PMC4207690; doi:10.1371/journal.pone.0110152)
Supplement: Table S5 — The association between Pseudomonas and semen quality in samples with and without relatively abundant Lactobacillus. (DOCX) [file pone.0110152.s005.docx]

**Table S5**. The association between *Pseudomonas* and semen quality in samples with and without relatively abundant *Lactobacillus*

|  | | Number of  normal samples | Number of  case samples | Fisher’s exact  p-value |
| --- | --- | --- | --- | --- |
| *Pseudomonas* relative abundant samples (35 #) ^a^ | | 19 | 16 | NA |
|  | - **with relative abundant   *Lactobacillus* (16 #) ^b^** | 12 | 4 | 0.041 |
|  | - **without relative abundant   *Lactobacillus* (19 #) ^c^** | 7 | 12 |  |

^a^ the proportion of *Pseudomonas* in sample greater than average proportion of *Pseudomonas*

^b^ the proportion of *Lactobacillus* in sample greater than average proportion of *Lactobacillus*

^c^ the proportion of *Lactobacillus* in sample less than average proportion of *Lactobacillus*
