# Supplementary material for: Bacterial Communities in Semen from Men of Infertile Couples: Metagenomic Sequencing Reveals Relationships of Seminal Microbiota to Semen Quality
Source: PLoS One. 2014 Oct 23;9(10):e110152. doi: 10.1371/journal.pone.0110152 (PMC4207690; doi:10.1371/journal.pone.0110152)
Supplement: Table S6 — Fisher's exact test and odds ratio of low semen quality rates comparing G1 and G3 to G2. (DOCX) [file pone.0110152.s006.docx]

**Table S6.** Fisher’s exact test and odds ratio of low semen quality rates comparing G1 and G3 to G2

| Semen bacterial community type | Number of  normal samples | Number of  case samples | p value of  Fisher’s exact test | odds ratio |
| --- | --- | --- | --- | --- |
| G2 | 29 | 13 | *ref.* | *ref.* |
| G1 | 5 | 12 | 8.40E-03 | 5.19 |
| G3 | 2 | 8 | 9.38E-03 | 8.52 |
