# Supplementary material for: Bacterial Communities in Semen from Men of Infertile Couples: Metagenomic Sequencing Reveals Relationships of Seminal Microbiota to Semen Quality
Source: PLoS One. 2014 Oct 23;9(10):e110152. doi: 10.1371/journal.pone.0110152 (PMC4207690; doi:10.1371/journal.pone.0110152)
Supplement: Table S7 — Genera of bacteria significantly abundant in samples with normal clinical value. (DOCX) [file pone.0110152.s007.docx]

**Table S7.** Genera of bacteria significantly abundant in samples with normal clinical value

| Clinical  criteria | Genus | Tax_ID | U-test p.value | Adj.p | normal mean / abnormal mean | normal mean ± SD | abnormal mean ± SD |
| --- | --- | --- | --- | --- | --- | --- | --- |
| Sperm  concentration | *Lactobacillus* | 1578 | 0.0013 | **0.03146273** | 2.70 | 2.5e-01 ± 2.5e-02 | 9.2e-02 ± 3.7e-02 |
|  | *Atopobium* | 1380 | 0.0180 | 0.1646898 | 3.33 | 7.0e-03 ± 1.4e-03 | 2.1e-03 ± 1.2e-03 |
|  | *Gardnerella* | 2701 | 0.0001 | **0.003138777** | 6.67 | 6.6e-02 ± 1.2e-02 | 1.0e-02 ± 6.0e-03 |
| Motility | *Gardnerella* | 2701 | 0.0048 | 0.253334 | 3.03 | 6.6e-02 ± 1.2e-02 | 2.2e-02 ± 9.0e-03 |
|  | *Anaerococcus* | 165779 | 0.0368 | 0.6499375 | 4.55 | 5.0e-03 ± 2.0e-03 | 1.1e-03 ± 4.9e-04 |
|  | *Staphylococcus* | 1279 | 0.0280 | 0.6499375 | 4.76 | 2.1e-02 ± 6.6e-03 | 4.4e-03 ± 1.4e-03 |
| Kruger’s strict morphology | *Lactobacillus* | 1578 | 0.0089 | 0.1363437 | 1.52 | 2.5e-01 ± 2.5e-02 | 1.6e-01 ± 2.8e-02 |
|  | *Gardnerella* | 2701 | 0.0008 | **0.03834393** | 2.33 | 6.6e-02 ± 1.2e-02 | 2.8e-02 ± 6.0e-03 |
| Antisperm antibody (IgA) | *Gardnerella* | 2701 | 0.0228 | 0.6721009 | 2.44 | 6.6e-02 ± 1.2e-02 | 2.7e-02 ± 1.4e-02 |
| Atypical | *Lactobacillus* | 1578 | 0.0300 | 0.3055766 | 2.00 | 2.5e-01 ± 2.5e-02 | 1.2e-01 ± 5.5e-02 |
|  | *Flexibacter* | 992 | 0.0397 | NA | 2.33 | 1.8e-03 ± 3.0e-04 | 7.7e-04 ± 3.6e-04 |
|  | *Gardnerella* | 2701 | 0.0101 | 0.1722180 | 4.55 | 6.6e-02 ± 1.2e-02 | 1.5e-02 ± 6.2e-03 |
| Leucocytes | *Pedobacter* | 84567 | 0.0161 | NA | 1.61 | 2.0e-03 ± 2.5e-04 | 1.2e-03 ± 4.2e-04 |
|  | *Atopobium* | 1380 | 0.0337 | 0.1759226 | 1.75 | 7.0e-03 ± 1.4e-03 | 4.0e-03 ± 2.1e-03 |
|  | *Lactobacillus* | 1578 | 0.0064 | 0.1362557 | 1.85 | 2.5e-01 ± 2.5e-02 | 1.3e-01 ± 4.0e-02 |
|  | *Propionibacterium* | 1743 | 0.0174 | 0.1362557 | 2.17 | 9.4e-03 ± 1.6e-03 | 4.4e-03 ± 1.2e-03 |
|  | *Gardnerella* | 2701 | 0.0027 | 0.1268140 | 3.13 | 6.6e-02 ± 1.2e-02 | 2.1e-02 ± 6.1e-03 |
|  | *Thermus* | 270 | 0.0195 | 0.1362557 | 843 | 4.3e-03 ± 1.9e-03 | 5.1e-06 ± 5.1e-06 |

Adj.p = adjust p value with FDR<0.05 using adaptive Benjamini-Hochberg method;

normal mean = average proportion of a genus in samples with normal clinical value;

abnormal mean = average proportion of a genus in samples with abnormal clinical value;

SD = standard deviation;

NA = not collected for calculating Adj.p due to the proportion of the genus less than 0.25%;
