# Supplementary material for: Bacterial Communities in Semen from Men of Infertile Couples: Metagenomic Sequencing Reveals Relationships of Seminal Microbiota to Semen Quality
Source: PLoS One. 2014 Oct 23;9(10):e110152. doi: 10.1371/journal.pone.0110152 (PMC4207690; doi:10.1371/journal.pone.0110152)
Supplement: Table S8 — Genera of bacteria significantly abundant in samples with abnormal clinical value. (DOCX) [file pone.0110152.s008.docx]

**Table S8.** Genera of bacteria significantly abundant in samples with abnormal clinical value

| Clinical  criteria | Genus | Tax_ID | U-test p.value | Adj.p | abnormal mean / normal mean | normal mean ± SD | abnormal mean ± SD |
| --- | --- | --- | --- | --- | --- | --- | --- |
| Semen Volume | *Shewanella* | 22 | 0.0418 | NA | 2.06 | 1.3e-03 ± 4.1e-04 | 2.6e-03 ± 9.9e-04 |
| Sperm  concentration | *Rubrimonas* | 89523 | 0.0036 | NA | ∞ | 0.0e+00 ± 0.0e+00 | 1.6e-03 ± 1.6e-03 |
|  | *Sphingobium* | 165695 | 0.0019 | NA | 4.31 | 9.2e-04 ± 4.2e-04 | 4.0e-03 ± 1.5e-03 |
|  | *Brevibacterium* | 1696 | 0.0147 | 0.1646898 | 2.59 | 2.0e-03 ± 7.1e-04 | 5.3e-03 ± 1.6e-03 |
|  | *Microbacterium* | 33882 | 0.0202 | 0.1646898 | 2.59 | 4.2e-03 ± 1.5e-03 | 1.1e-02 ± 3.7e-03 |
|  | *Aggregatibacter* | 416916 | 0.0263 | NA | 2.48 | 7.1e-04 ± 5.1e-04 | 1.7e-03 ± 7.8e-04 |
|  | *Burkholderia* | 32008 | 0.0463 | 0.3240602 | 2.39 | 3.7e-03 ± 1.3e-03 | 9.0e-03 ± 3.0e-03 |
|  | *Polaromonas* | 52972 | 0.0112 | NA | 2.29 | 6.4e-04 ± 1.5e-04 | 1.5e-03 ± 3.6e-04 |
|  | *Comamonas* | 283 | 0.0053 | **0.08674954** | 1.98 | 3.3e-03 ± 5.0e-04 | 6.6e-03 ± 1.1e-03 |
|  | *Pasteurella* | 745 | 0.0270 | NA | 1.90 | 1.7e-03 ± 3.5e-04 | 3.2e-03 ± 7.6e-04 |
|  | *Dermacoccus* | 57495 | 0.0216 | NA | 1.89 | 9.2e-04 ± 3.5e-04 | 1.7e-03 ± 5.9e-04 |
| Kruger’s strict  morphology | *Varibaculum* | 184869 | 0.0127 | NA | 5.62 | 4.2e-04 ± 1.5e-04 | 2.3e-03 ± 1.0e-03 |
|  | *Mobiluncus* | 2050 | 0.0343 | 0.2100993 | 4.29 | 1.2e-03 ± 3.6e-04 | 5.0e-03 ± 2.2e-03 |
|  | *Peptoniphilus* | 162289 | 0.0357 | 0.2100993 | 2.33 | 4.5e-03 ± 1.4e-03 | 1.1e-02 ± 2.5e-03 |
|  | *Prevotella* | 838 | 0.0052 | 0.1186493 | 2.05 | 5.4e-02 ± 1.1e-02 | 1.1e-01 ± 1.8e-02 |
|  | *Campylobacter* | 194 | 0.0178 | 0.1638209 | 2.02 | 7.2e-03 ± 2.8e-03 | 1.4e-02 ± 4.4e-03 |
|  | *Dialister* | 39948 | 0.0395 | 0.2100993 | 1.90 | 5.5e-03 ± 1.4e-03 | 1.1e-02 ± 2.1e-03 |
|  | *Porphyromonas* | 836 | 0.0175 | 0.1638209 | 1.71 | 7.7e-03 ± 2.2e-03 | 1.3e-02 ± 2.8e-03 |
| Antisperm antibody (IgA) | *Sphingobium* | 165695 | 0.0215 | NA | 3.71 | 9.2e-04 ± 4.2e-04 | 3.4e-03 ± 1.6e-03 |
| Atypical | *Alloscardovia* | 419014 | 0.0028 | NA | 67.47 | 4.1e-05 ± 4.1e-05 | 2.8e-03 ± 2.5e-03 |
|  | *Varibaculum* | 184869 | 0.0025 | NA | 4.30 | 4.2e-04 ± 1.5e-04 | 1.8e-03 ± 6.1e-04 |
|  | *Haemophilus* | 724 | 0.0496 | 0.4216327 | 4.28 | 1.7e-02 ± 7.5e-03 | 7.1e-02 ± 4.4e-02 |
|  | *Rubrobacter* | 42255 | 0.0359 | NA | 4.23 | 3.2e-04 ± 9.0e-05 | 1.4e-03 ± 5.7e-04 |
|  | *Peptoniphilus* | 162289 | 0.0181 | 0.2311795 | 3.14 | 4.5e-03 ± 1.4e-03 | 1.4e-02 ± 5.9e-03 |
|  | *Campylobacter* | 194 | 0.0098 | 0.1722180 | 2.51 | 7.2e-03 ± 2.8e-03 | 1.8e-02 ± 9.2e-03 |
|  | *Prevotella* | 838 | 0.0018 | **0.09409667** | 2.24 | 5.4e-02 ± 1.1e-02 | 1.2e-01 ± 2.1e-02 |
|  | *Aggregatibacter* | 416916 | 0.0161 | NA | 2.14 | 7.1e-04 ± 5.1e-04 | 1.5e-03 ± 7.4e-04 |
| Leucocytes | *Aggregatibacter* | 416916 | 0.0114 | NA | 6.53 | 7.1e-04 ± 5.1e-04 | 4.6e-03 ± 2.7e-03 |
|  | *Negativicoccus* | 909928 | 0.0018 | NA | 4.53 | 5.4e-04 ± 2.4e-04 | 2.5e-03 ± 6.5e-04 |
|  | *Sphingobium* | 165695 | 0.0198 | NA | 2.90 | 9.2e-04 ± 4.2e-04 | 2.7e-03 ± 9.6e-04 |
|  | *Campylobacter* | 194 | 0.0107 | 0.1362557 | 2.52 | 7.2e-03 ± 2.8e-03 | 1.8e-02 ± 6.5e-03 |
|  | *Haemophilus* | 724 | 0.0310 | 0.1759226 | 2.47 | 1.7e-02 ± 7.5e-03 | 4.1e-02 ± 2.0e-02 |
|  | *Brevibacterium* | 1696 | 0.0203 | 0.1362557 | 2.01 | 2.0e-03 ± 7.1e-04 | 4.1e-03 ± 1.3e-03 |
|  | *Prevotella* | 838 | 0.0126 | 0.1362557 | 1.88 | 5.4e-02 ± 1.1e-02 | 1.0e-01 ± 2.0e-02 |
|  | *Dermacoccus* | 57495 | 0.0097 | NA | 1.86 | 9.2e-04 ± 3.5e-04 | 1.7e-03 ± 4.7e-04 |
|  | *Pasteurella* | 745 | 0.0405 | NA | 1.52 | 1.7e-03 ± 3.5e-04 | 2.5e-03 ± 5.1e-04 |

Adj.p = adjust p value with FDR<0.05 using adaptive Benjamini-Hochberg method;

normal mean = average proportion of a genus in samples with normal clinical value;

abnormal mean = average proportion of a genus in samples with abnormal clinical value;

SD = standard deviation;

NA = not collected for calculating Adj.p due to the proportion of the genus less than 0.25%;
