# Supplementary material for: Bacterial Communities in Semen from Men of Infertile Couples: Metagenomic Sequencing Reveals Relationships of Seminal Microbiota to Semen Quality
Source: PLoS One. 2014 Oct 23;9(10):e110152. doi: 10.1371/journal.pone.0110152 (PMC4207690; doi:10.1371/journal.pone.0110152)
Supplement: Table S9 — Species of bacteria significantly abundant in samples with normal clinical value. (DOCX) [file pone.0110152.s009.docx]

**Table S9.** Species of bacteria significantly abundant in samples with normal clinical value

| Clinical  criteria | Specie | Tax_ID | U-test p.value | Adj.p | normal mean /  abnormal mean | normal mean ± SD | abnormal mean ± SD |
| --- | --- | --- | --- | --- | --- | --- | --- |
| Sperm  concentration | uncultured Lactobacillus sp. | 153152 | 0.0009 | **0.01888572** | 3.03 | 3.2e-02 ± 3.5e-03 | 1.0e-02 ± 3.9e-03 |
|  | Pseudomonas sp. ps10-13 | 244615 | 0.0010 | NA | 3.23 | 1.0e-03 ± 1.7e-04 | 3.2e-04 ± 1.6e-04 |
|  | Atopobium vaginae | 82135 | 0.0066 | **0.08374556** | 3.70 | 5.6e-03 ± 1.1e-03 | 1.5e-03 ± 1.1e-03 |
|  | Lactobacillus crispatus | 47770 | 0.0075 | **0.08374556** | 5.26 | 3.8e-02 ± 8.9e-03 | 7.3e-03 ± 4.0e-03 |
|  | Lactobacillus acidophilus | 1579 | 0.0214 | 0.174197 | 6.25 | 1.2e-02 ± 2.8e-03 | 1.9e-03 ± 9.7e-04 |
|  | uncultured Gardnerella sp. | 293424 | 0.0001 | **0.004394731** | 6.25 | 4.5e-02 ± 9.7e-03 | 7.3e-03 ± 4.8e-03 |
|  | Gardnerella vaginalis | 2702 | 0.0001 | **0.004394731** | 7.14 | 2.0e-02 ± 4.2e-03 | 2.7e-03 ± 1.2e-03 |
| Motility | uncultured Staphylococcus sp. | 189668 | 0.0432 | 0.7124139 | 4.76 | 1.6e-02 ± 4.9e-03 | 3.3e-03 ± 1.0e-03 |
|  | Gardnerella vaginalis | 2702 | 0.0010 | **0.07346134** | 5.56 | 2.0e-02 ± 4.2e-03 | 3.7e-03 ± 1.5e-03 |
|  | uncultured Anaerococcus sp. | 293428 | 0.0354 | NA | 7.69 | 1.5e-03 ± 6.0e-04 | 2.0e-04 ± 1.2e-04 |
| Leucocytes | Gardnerella vaginalis | 2702 | 0.0009 | **0.0517445** | 4.17 | 2.0e-02 ± 4.2e-03 | 4.9e-03 ± 1.5e-03 |
|  | Lactobacillus crispatus | 47770 | 0.0055 | 0.1112540 | 7.69 | 3.8e-02 ± 8.9e-03 | 4.8e-03 ± 1.8e-03 |
|  | Lactobacillus acidophilus | 1579 | 0.0070 | 0.1112540 | 9.09 | 1.2e-02 ± 2.8e-03 | 1.3e-03 ± 5.2e-04 |
|  | Lactobacillus gallinarum | 52242 | 0.0449 | NA | 11.11 | 1.9e-03 ± 4.9e-04 | 1.8e-04 ± 7.6e-05 |
|  | Thermus scotoductus | 37636 | 0.0494 | NA | ∞ | 1.4e-03 ± 6.3e-04 | 0.0e+00 ± 0.0e+00 |
|  | uncultured Thermus sp. | 157149 | 0.0338 | NA | ∞ | 1.2e-03 ± 5.0e-04 | 0.0e+00 ± 0.0e+00 |
| Antisperm antibody (IgA) | Gardnerella vaginalis | 2702 | 0.0093 | 0.3469474 | 4.00 | 2.0e-02 ± 4.2e-03 | 5.0e-03 ± 2.2e-03 |
|  | Pseudomonas sp. ps10-13 | 244615 | 0.0041 | NA | 4.00 | 1.0e-03 ± 1.7e-04 | 2.6e-04 ± 1.3e-04 |
|  | Lactobacillus crispatus | 47770 | 0.0442 | 0.3469474 | 4.17 | 3.8e-02 ± 8.9e-03 | 8.9e-03 ± 5.4e-03 |
|  | Atopobium vaginae | 82135 | 0.0113 | 0.3469474 | 8.33 | 5.6e-03 ± 1.1e-03 | 6.5e-04 ± 3.8e-04 |
|  | Streptococcus agalactiae | 1311 | 0.0496 | 0.3469474 | 14.29 | 3.6e-03 ± 1.8e-03 | 2.7e-04 ± 2.0e-04 |
| Atypical | Pseudomonas sp. G1013 | 410949 | 0.0341 | NA | 3.45 | 1.7e-03 ± 3.4e-04 | 4.9e-04 ± 2.2e-04 |
|  | Pseudomonas sp. ps10-13 | 244615 | 0.0142 | NA | 3.70 | 1.0e-03 ± 1.7e-04 | 2.8e-04 ± 1.2e-04 |
|  | Gardnerella vaginalis | 2702 | 0.0169 | 0.1062832 | 4.35 | 2.0e-02 ± 4.2e-03 | 4.6e-03 ± 2.2e-03 |
|  | uncultured Gardnerella sp. | 293424 | 0.0110 | **0.09957225** | 4.55 | 4.5e-02 ± 9.7e-03 | 1.0e-02 ± 4.3e-03 |
|  | Haemophilus parahaemolyticus | 735 | 0.0321 | NA | ∞ | 1.2e-03 ± 5.5e-04 | 0.0e+00 ± 0.0e+00 |
| Kruger’s strict  morphology | Lactobacillus crispatus | 47770 | 0.0096 | 0.1013307 | 3.33 | 3.8e-02 ± 8.9e-03 | 1.1e-02 ± 3.1e-03 |
|  | Lactobacillus acidophilus | 1579 | 0.0220 | 0.1383824 | 3.57 | 1.2e-02 ± 2.8e-03 | 3.2e-03 ± 8.3e-04 |

Adj.p = adjust p value with FDR<0.05 using adaptive Benjamini-Hochberg method;

normal mean = average proportion of a specie in samples with normal clinical value;

abnormal mean = average proportion of a specie in samples with abnormal clinical value;

SD = standard deviation;

NA = not collected for calculating Adj.p due to the proportion of the specie less than 0.25%;
