# Supplementary material for: Bacterial Communities in Semen from Men of Infertile Couples: Metagenomic Sequencing Reveals Relationships of Seminal Microbiota to Semen Quality
Source: PLoS One. 2014 Oct 23;9(10):e110152. doi: 10.1371/journal.pone.0110152 (PMC4207690; doi:10.1371/journal.pone.0110152)
Supplement: Table S10 — Species of bacteria significantly abundant in samples with abnormal clinical value. (DOCX) [file pone.0110152.s010.docx]

**Table S10.** Species of bacteria significantly abundant in samples with abnormal clinical value

| Clinical  criteria | Specie | Tax_ID | U-test p.value | Adj.p | abnormal mean / normal mean | normal mean ± SD | abnormal mean ± SD |
| --- | --- | --- | --- | --- | --- | --- | --- |
| Semen Volume | *Fusobacterium periodonticum* | 860 | 0.0223 | NA | 3.84 | 4.0e-04 ± 1.8e-04 | 1.5e-03 ± 6.1e-04 |
| Sperm  concentration | *Porphyromonas somerae* | 322095 | 0.0036 | NA | ∞ | 0.0e+00 ± 0.0e+00 | 1.1e-03 ± 1.1e-03 |
|  | *Rubrimonas sp. SL014B-28A2* | 690083 | 0.0036 | NA | ∞ | 0.0e+00 ± 0.0e+00 | 1.6e-03 ± 1.6e-03 |
|  | *Brevundimonas sp. SP1* | 470051 | 0.0407 | NA | 123.52 | 1.3e-05 ± 8.3e-06 | 1.6e-03 ± 1.5e-03 |
|  | *Actinomyces turicensis* | 131111 | 0.0407 | NA | 26.87 | 5.8e-05 ± 5.3e-05 | 1.6e-03 ± 1.5e-03 |
|  | *Haemophilus parainfluenzae* | 729 | 0.0414 | 0.2987654 | 4.98 | 3.8e-03 ± 2.4e-03 | 1.9e-02 ± 1.6e-02 |
|  | *Sphingobium estrogenivorans* | 399103 | 0.0054 | NA | 4.63 | 6.7e-04 ± 3.2e-04 | 3.1e-03 ± 1.2e-03 |
|  | *Arthrobacter sp. Zn12* | 1048300 | 0.0142 | 0.1317269 | 4.04 | 1.5e-03 ± 7.3e-04 | 6.1e-03 ± 2.6e-03 |
|  | *Microbacterium laevaniformans* | 36807 | 0.0069 | NA | 3.75 | 5.6e-04 ± 2.5e-04 | 2.1e-03 ± 7.9e-04 |
|  | *Microbacterium testaceum* | 2033 | 0.0070 | NA | 3.01 | 3.9e-04 ± 1.1e-04 | 1.2e-03 ± 4.0e-04 |
| Motility | *Porphyromonas somerae* | 322095 | 0.0146 | NA | ∞ | 0.0e+00 ± 0.0e+00 | 1.2e-03 ± 1.2e-03 |
|  | *uncultured Achromobacter sp.* | 182690 | 0.0319 | NA | 7.81 | 1.4e-04 ± 4.2e-05 | 1.1e-03 ± 7.6e-04 |
|  | *Arthrobacter sp. Zn12* | 1048300 | 0.0148 | 0.472741 | 3.55 | 1.5e-03 ± 7.3e-04 | 5.4e-03 ± 2.8e-03 |
| Kruger’s strict  morphology | *Prevotella nanceiensis* | 425941 | 0.0496 | NA | 7.02 | 1.5e-04 ± 8.2e-05 | 1.1e-03 ± 4.3e-04 |
|  | *Mobiluncus curtisii* | 2051 | 0.0189 | 0.1383824 | 5.92 | 7.7e-04 ± 2.6e-04 | 4.6e-03 ± 2.0e-03 |
|  | *Varibaculum cambriense* | 184870 | 0.0127 | NA | 5.62 | 4.2e-04 ± 1.5e-04 | 2.3e-03 ± 1.0e-03 |
|  | *Prevotella sp. BV3C7* | 1111129 | 0.0144 | NA | 4.59 | 2.9e-04 ± 1.0e-04 | 1.3e-03 ± 3.7e-04 |
| Antisperm antibody (IgA) | *Prevotella melaninogenica* | 28132 | 0.0419 | NA | 10.88 | 3.8e-04 ± 1.9e-04 | 4.1e-03 ± 3.4e-03 |
|  | *Arthrobacter sp. Zn12* | 1048300 | 0.0363 | 0.3469474 | 4.19 | 1.5e-03 ± 7.3e-04 | 6.3e-03 ± 3.2e-03 |
|  | *uncultured Neisseria sp.* | 237778 | 0.0351 | 0.3469474 | 3.99 | 4.1e-03 ± 1.8e-03 | 1.7e-02 ± 7.6e-03 |
|  | *Sphingobium estrogenivorans* | 399103 | 0.0231 | NA | 3.81 | 6.7e-04 ± 3.2e-04 | 2.6e-03 ± 1.3e-03 |
| Atypical | *Alloscardovia omnicolens* | 419015 | 0.0028 | NA | 67.47 | 4.1e-05 ± 4.1e-05 | 2.8e-03 ± 2.5e-03 |
|  | *Citrobacter koseri* | 545 | 0.0236 | NA | 53.18 | 6.3e-05 ± 6.3e-05 | 3.4e-03 ± 2.9e-03 |
|  | *Anaerococcus murdochii* | 411577 | 0.0003 | NA | 26.66 | 1.5e-04 ± 9.2e-05 | 4.1e-03 ± 2.7e-03 |
|  | *Peptoniphilus asaccharolyticus* | 1258 | 0.0281 | NA | 6.65 | 2.3e-04 ± 5.6e-05 | 1.5e-03 ± 7.9e-04 |
|  | *Streptococcus anginosus* | 1328 | 0.0072 | **0.09957225** | 5.95 | 1.8e-03 ± 9.3e-04 | 1.1e-02 ± 4.7e-03 |
|  | *Finegoldia sp. BV3C29* | 1111124 | 0.0353 | NA | 5.81 | 5.0e-04 ± 1.3e-04 | 2.9e-03 ± 1.3e-03 |
|  | *Catonella sp. oral clone AH153* | 135029 | 0.0209 | NA | 5.33 | 2.3e-04 ± 1.8e-04 | 1.3e-03 ± 7.5e-04 |
|  | *Prevotella sp. BV3C7* | 1111129 | 0.0212 | NA | 5.33 | 2.9e-04 ± 1.0e-04 | 1.6e-03 ± 8.5e-04 |
|  | *uncultured Peptoniphilus sp.* | 254354 | 0.0143 | NA | 5.28 | 7.1e-04 ± 1.6e-04 | 3.8e-03 ± 1.8e-03 |
|  | *Varibaculum cambriense* | 184870 | 0.0025 | NA | 4.30 | 4.2e-04 ± 1.5e-04 | 1.8e-03 ± 6.1e-04 |
|  | *Prevotella bivia* | 28125 | 0.0030 | **0.09957225** | 4.19 | 1.1e-02 ± 4.8e-03 | 4.5e-02 ± 1.9e-02 |
|  | *Porphyromonas uenonis* | 281920 | 0.0289 | NA | 3.89 | 8.8e-04 ± 2.7e-04 | 3.4e-03 ± 1.5e-03 |
|  | *Haemophilus pittmaniae* | 249188 | 0.0044 | **0.09957225** | 3.61 | 4.8e-03 ± 3.5e-03 | 1.7e-02 ± 1.1e-02 |
|  | *Actinomyces sp. 'Smarlab BioMol-2300463'* | 225907 | 0.0272 | NA | 3.37 | 3.5e-04 ± 2.0e-04 | 1.2e-03 ± 8.6e-04 |
| Leucocytes | *Veillonella ratti* | 103892 | 0.0465 | NA | ∞ | 0.0e+00 ± 0.0e+00 | 6.5e-03 ± 6.3e-03 |
|  | *Corynebacterium sp. 1119* | 651865 | 0.0012 | NA | 6.08 | 4.8e-04 ± 3.9e-04 | 2.9e-03 ± 1.9e-03 |
|  | *Corynebacterium sp. NML94-0264* | 702960 | 0.0095 | NA | 6.06 | 3.2e-04 ± 2.6e-04 | 2.0e-03 ± 1.3e-03 |
|  | *Corynebacterium sp. 31595* | 288147 | 0.0102 | NA | 5.90 | 3.6e-04 ± 3.2e-04 | 2.1e-03 ± 1.3e-03 |
|  | *Actinomyces sp. 'Smarlab BioMol-2300463'* | 225907 | 0.0442 | NA | 5.49 | 3.5e-04 ± 2.0e-04 | 1.9e-03 ± 1.1e-03 |
|  | *Corynebacterium sp. NML96-0085* | 702963 | 0.0113 | NA | 5.06 | 2.5e-04 ± 2.1e-04 | 1.2e-03 ± 9.7e-04 |
|  | *Negativicoccus succinicivorans* | 620903 | 0.0018 | NA | 4.53 | 5.4e-04 ± 2.4e-04 | 2.5e-03 ± 6.5e-04 |
|  | *uncultured Campylobacter sp.* | 218934 | 0.0323 | 0.1643388 | 3.55 | 2.1e-03 ± 7.6e-04 | 7.3e-03 ± 3.5e-03 |

Adj.p = adjust p value with FDR<0.05 using adaptive Benjamini-Hochberg method;

normal mean = average proportion of a specie in samples with normal clinical value;

abnormal mean = average proportion of a specie in samples with abnormal clinical value;

SD = standard deviation;

NA = not collected for calculating Adj.p due to the proportion of the specie less than 0.25%;
