# Supplementary material for: Bacterial Communities in Semen from Men of Infertile Couples: Metagenomic Sequencing Reveals Relationships of Seminal Microbiota to Semen Quality
Source: PLoS One. 2014 Oct 23;9(10):e110152. doi: 10.1371/journal.pone.0110152 (PMC4207690; doi:10.1371/journal.pone.0110152)
Supplement: Table S11 — Genera of bacteria significantly associated with CASA criteria. (DOCX) [file pone.0110152.s011.docx]

**Table S11.** Genera of bacteria significantly associated with CASA criteria

| CASA criteria | Genus | Correlation | P value | average proportion of genus in samples |
| --- | --- | --- | --- | --- |
| Elongation | Brevibacterium | -0.590817925 | 2.35E-10 | 0.002773812 |
| Elongation | Dermacoccus | -0.568616563 | 1.51E-09 | 0.001153206 |
| Elongation | Sphingobium | -0.546612224 | 8.35E-09 | 0.001615955 |
| Elongation | Burkholderia | -0.523073336 | 4.57E-08 | 0.004736716 |
| Elongation | Curvibacter | -0.609078036 | 4.57E-11 | 0.002372698 |
| Elongation | Pelomonas | -0.500878415 | 2.02E-07 | 0.001734369 |
| Elongation | Variovorax | -0.566253594 | 1.82E-09 | 0.001677096 |
| Area | Brevibacterium | 0.547131091 | 8.03E-09 | 0.002773812 |
| Area | Dermacoccus | 0.602108031 | 8.64E-11 | 0.001153206 |
| Area | Curvibacter | 0.557511826 | 3.63E-09 | 0.002372698 |
| Area | Variovorax | 0.590302537 | 2.46E-10 | 0.001677096 |
