# Supplementary material for: Bacterial Communities in Semen from Men of Infertile Couples: Metagenomic Sequencing Reveals Relationships of Seminal Microbiota to Semen Quality
Source: PLoS One. 2014 Oct 23;9(10):e110152. doi: 10.1371/journal.pone.0110152 (PMC4207690; doi:10.1371/journal.pone.0110152)
Supplement: Table S12 — Species of bacteria significantly associated with CASA criteria. (DOCX) [file pone.0110152.s012.docx]

**Table S12.** Species of bacteria significantly associated with CASA criteria

| CASA criteria | specie | Correlation | P value | average proportion of specie in samples |
| --- | --- | --- | --- | --- |
| Elongation | Brevibacterium sanguinis | -0.585680259 | 3.66E-10 | 0.001290006 |
| Elongation | Dermacoccus sp. HOR6-4 | -0.576954207 | 7.62E-10 | 0.001146693 |
| Elongation | Arthrobacter sanguinis | -0.518392352 | 6.30E-08 | 0.001749201 |
| Elongation | uncultured Atopobium sp. | 0.53906095 | 1.46E-08 | 0.001056987 |
| Elongation | Lactobacillus crispatus | 0.508410942 | 1.23E-07 | 0.021404268 |
| Elongation | Sphingobium estrogenivorans | -0.576383963 | 7.99E-10 | 0.001253492 |
| Elongation | Burkholderia cepacia | -0.54854597 | 7.22E-09 | 0.00125239 |
| Elongation | Burkholderia multivorans | -0.605928193 | 6.10E-11 | 0.001983053 |
| Elongation | uncultured Curvibacter sp. | -0.649193599 | 8.43E-13 | 0.001110896 |
| Elongation | Delftia acidovorans | -0.517007593 | 6.93E-08 | 0.001776237 |
| Elongation | uncultured Variovorax sp. | -0.606225969 | 5.94E-11 | 0.00111658 |
| Elongation | Pasteurella testudinis | -0.532179768 | 2.40E-08 | 0.001734158 |
| Elongation | Pseudomonas sp. N10-6zhy | -0.567172953 | 1.69E-09 | 0.001007135 |
| Area | Brevibacterium sanguinis | 0.569540051 | 1.40E-09 | 0.001290006 |
| Area | Dermacoccus sp. HOR6-4 | 0.603303443 | 7.75E-11 | 0.001146693 |
| Area | Arthrobacter sp. Zn12 | 0.522529222 | 4.74E-08 | 0.002793514 |
| Area | Sphingobium estrogenivorans | 0.526518138 | 3.59E-08 | 0.001253492 |
| Area | Burkholderia cepacia | 0.518177795 | 6.40E-08 | 0.00125239 |
| Area | Burkholderia multivorans | 0.601167278 | 9.40E-11 | 0.001983053 |
| Area | uncultured Curvibacter sp. | 0.588497434 | 2.87E-10 | 0.001110896 |
| Area | uncultured Variovorax sp. | 0.598176849 | 1.23E-10 | 0.00111658 |
| Area | Klebsiella pneumoniae | 0.503523041 | 1.70E-07 | 0.00247844 |
| Area | Pasteurella testudinis | 0.53207356 | 2.42E-08 | 0.001734158 |
| Area | Pseudomonas sp. N10-6zhy | 0.583196759 | 4.52E-10 | 0.001007135 |
