# Supplementary material for: Bacterial Communities in Semen from Men of Infertile Couples: Metagenomic Sequencing Reveals Relationships of Seminal Microbiota to Semen Quality
Source: PLoS One. 2014 Oct 23;9(10):e110152. doi: 10.1371/journal.pone.0110152 (PMC4207690; doi:10.1371/journal.pone.0110152)
Supplement: Table S13 — Performance classification of microbiome community types using four bacteria, Lactobacillus, Prevotella, Pseudomonas and Haemophilus. (DOCX) [file pone.0110152.s013.docx]

**Table S13**. Performance classification of microbiome community types using four bacteria, *Lactobacillus*, *Prevotella*, *Pseudomonas* and *Haemophilus*

|  | G1 | G2 | G3 | Weighted Avg. |
| --- | --- | --- | --- | --- |
| TP Rate | 0.96 | 0.964 | 0.875 | 0.948 |
| FP Rate | 0.028 | 0.049 | 0.013 | 0.037 |
| Precision | 0.923 | 0.964 | 0.933 | 0.948 |
| Recall | 0.96 | 0.964 | 0.875 | 0.948 |
| F-Measure | 0.941 | 0.964 | 0.903 | 0.948 |
| AUC | 0.954 | 0.953 | 0.913 | 0.946 |

TP: true positive, FP: false positive, Precision: TP/(TP+FP), Recall=TP/(TP+FN), FN: false negative, F-Measure= 2*Precision*Recall/(Precision+Recall), AUC: area under the ROC curve, ROC: receiver operating characteristic
